# The pyruvate generator is a common phenomenon in mitochondria from different rat and mouse brain regions

**DOI:** 10.1002/1873-3468.70370

**Published:** 2026-05-23

**Authors:** Grazyna Debska‐Vielhaber, Niki Karavasili, Matthias Kunz, Zemfira Gizatullina, Timur Gainutdinov, Stefan Vielhaber, Wolfram S. Kunz, Frank N. Gellerich

**Affiliations:** ^1^ Department of Neurology Otto‐von‐Guericke University Magdeburg Germany; ^2^ Department of General Visceral and Minimal Invasive Surgery at Helios Hospital Emil von Behring Berlin Germany; ^3^ Department of Cardiology and Angiology Otto‐von‐Guericke University Magdeburg Germany; ^4^ Tatarstan Academy of Sciences Kazan Russia; ^5^ Department of Epileptology University of Bonn Germany

**Keywords:** extra‐mitochondrial calcium, glutamate respiration, malate–aspartate shuttle, oxidative phosphorylation, rat brain mitochondria

## Abstract

The finding of the pyruvate generator (‘mitochondrial gas pedal’) arose from the observation that cytosolic Ca^2+^ accelerates glutamate‐driven respiration. Here, we show that glutamate respiration of isolated rat brainstem mitochondria appears to be insensitive to extra‐mitochondrial Ca^2^^+^. This raises the question: Do these mitochondria lack a pyruvate generator, or is its detection masked? By reconstituting the complete malate–aspartate shuttle (MAS), we demonstrate that brainstem mitochondria possess a pyruvate generator, just like mitochondria from other brain regions. Direct measurement, however, is hindered by the high rate of Ca^2+^‐insensitive glutamate utilization by glial mitochondria. We therefore conclude that the pyruvate generator is a universal mechanism in all tissues that contain a functional MAS and pyruvate‐generating enzymes.

## Graphical abstract

The pyruvate generator, which causes activation of respiration by extra‐mitochondrial Ca^2+^, is also present and functional in rat brainstem mitochondria, as it is in other brain regions. This finding is confirmed by experiments with a fully reconstituted malate–aspartate shuttle (MAS). A simple measurement of Ca^2+^‐stimulated glutamate‐dependent respiration in these mitochondria is compromised by their Ca^2+^‐insensitive glutamate utilization.

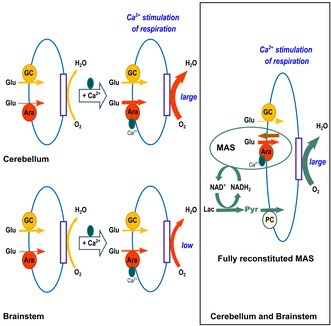

## Abbreviations

**AOA**, amino‐acetic acid

**CAT**, carboxyatractyloside

**CIN**, cinnamate

**CNS**, central nervous system

**MAS**, malate–aspartate shuttle

**MCU**, mitochondrial calcium uniporter

**MCU‐KO**, MCU‐knockout

**OXPHOS**, oxidative phosphorylation

**RuR**, ruthenium red

Cytosolic calcium (Ca^2+^_cyt_) plays a fundamental role in cell physiology, regulating cellular energy metabolism (ATP consumption and ATP production under various loading conditions) as well as other important intracellular processes, such as gene expression, muscle contraction, neurotransmitter release, and cell death [1, 2, 3, 4, 5, 6].

It is generally accepted that the rate of oxidative phosphorylation (OXPHOS) is regulated by Ca^2+^_cyt_ [7]. This classical mechanism involves Ca^2+^ entry into mitochondria via the mitochondrial calcium uniporter (MCU), which activates essential mitochondrial dehydrogenases: pyruvate dehydrogenase (PDH), α‐ketoglutarate dehydrogenase (α‐KGDH), and isocitrate dehydrogenase (ICDH) [8, 9]. A long‐standing issue with this mechanism is the low affinity of the MCU for Ca^2+^ (*K*_m_ = 10–20 μm) [10, 11], consequently, the MCU is essentially inactive at normal physiological Ca^2+^_cyt_ concentrations (between 100 and 700 nm) and becomes active only when cytosolic Ca^2+^ is markedly elevated [12].

The fact that mice lacking mitochondrial calcium uniporter (MCU‐KO) are viable under laboratory conditions and display no alteration in basal metabolism demonstrates that OXPHOS regulation does not require direct Ca^2+^ entry into the mitochondrial matrix. However, MCU‐KO mice exhibit a significant reduction in maximum skeletal muscle strength and impaired physical performance [13], which may limit their survival in natural conditions. It therefore appears that an alternative mechanism for OXPHOS regulation—the pyruvate generator—is operating, which is controlled by physiological Ca^2+^ concentration in the cytosol. According to the classical model, mitochondrial pyruvate‐dependent respiration should increase as cytosolic Ca^2+^ rises. However, we previously reported that the rate of pyruvate‐dependent respiration in isolated heart and brain mitochondria is already close to maximal rate (~85% *V*_max_) even in the absence of added Ca^2+^. The addition of extra‐mitochondrial Ca^2+^ can raise the respiration rate by a most of ~15% [14]. In contrast, glutamate‐dependent respiration of brain mitochondria is very low without Ca^2+^, and the addition of extra‐mitochondrial Ca^2+^ accelerates this respiration by a factor of 2–3, even when mitochondrial calcium uptake is inhibited with ruthenium red (RuR) or mitochondria are isolated from MCU‐KO mice [14, 15].

**Fig. 1:**
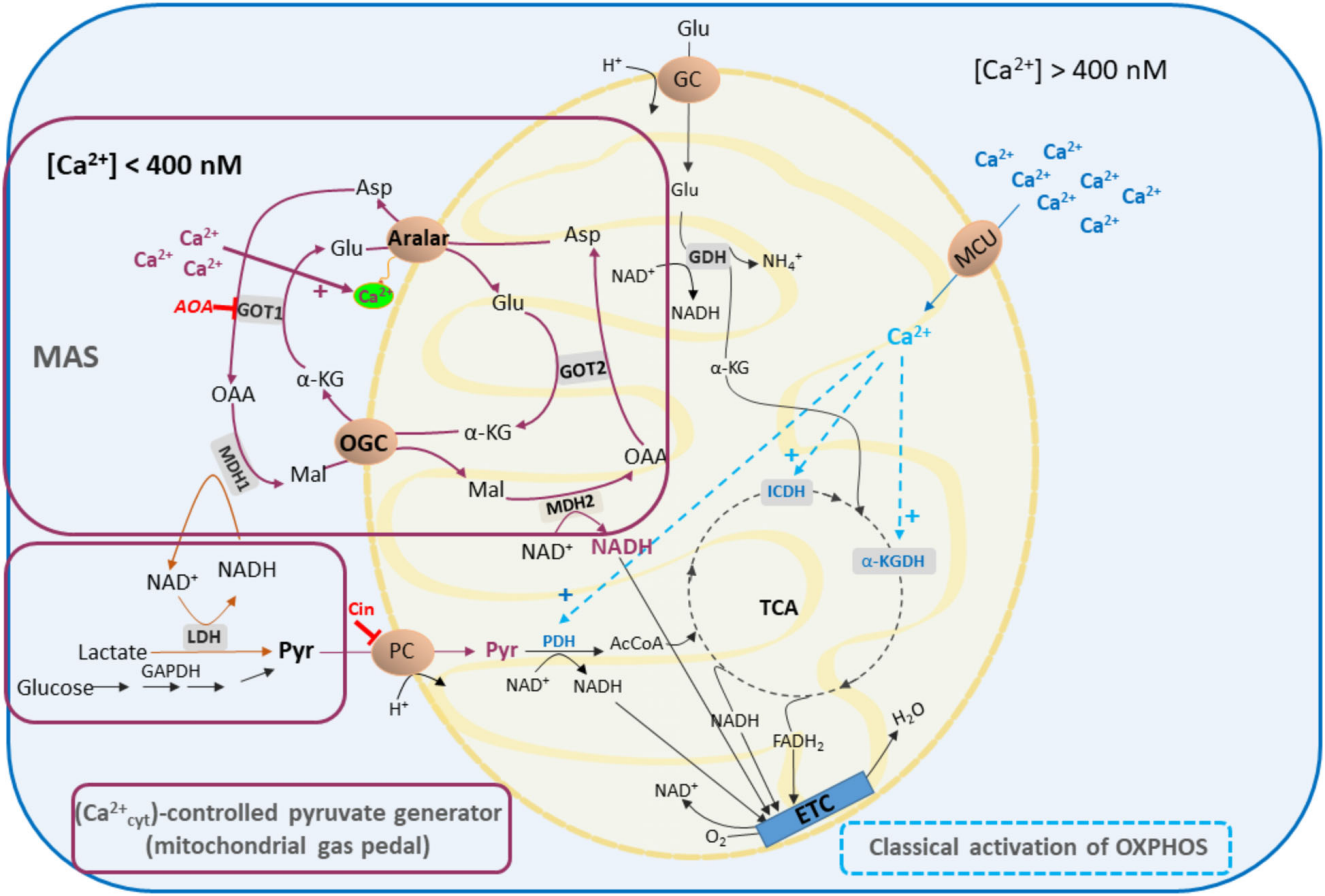
Schematic illustration of the regulation of oxidative phosphorylation (OXPHOS) by calcium ions. Red boxes denote the pyruvate generator (mitochondrial ‘gas pedal’), which consists of *pure* malate–aspartate shuttle (MAS) together with pyruvate‐producing enzymes. This system is activated by low cytoplasmic Ca^2+^ concentrations that do not engage the mitochondrial calcium uniporter (MCU). The MAS comprises: the Ca^2+^‐sensitive aralar (AGC1) and the oxoglutarate carrier (OGC)—antiporters that import glutamate and malate into matrix while exporting aspartate and α‐ketoglutarate, two cytosolic enzymes—glutamate‐oxaloacetate transaminase (GOT1) and malate dehydrogenase (MDH1), as well as two mitochondrial isoforms—GOT2 and MDH2. In the cytosol, GOT1 and MDH1 regenerate the MAS substrates, so substrate concentrations remain constant during shuttle activity. The cytosolic part of MAS oxidizes NADH to NAD^+^, whereas the mitochondrial part reduces NAD^+^ to NADH. Consequently, the MAS transfers only reducing equivalents (hydride ions) derived from lactate into the mitochondria without moving carbon skeletons. This tight coupling of *pure* MAS with oxidative pyruvate formation—often referred to as a complete MAS—constitutes an efficient, Ca^2+^_cyt_‐regulated pyruvate generator. Pyruvate generated in the cytosol is imported into matrix with high efficiency by the pyruvate carrier (PC), where it enters the tricarboxylic‐acid (TCA) cycle and ultimately supplies reducing equivalents to the electron‐transport chain (ETC). Glutamate can also enter the matrix independently of Ca^2+^ via the glutamate carrier (GC). Inside the matrix, glutamate dehydrogenase (GDH) converts glutamate to α‐ketoglutarate, which then feeds the TCA cycle. Blue dotted lines depict the classical OXPHOS activation pathway: Ca^2+^ uptake through MCU followed by activation of mitochondrial dehydrogenases—isocitrate dehydrogenase (ICDH), α‐ketoglutarate dehydrogenase (α‐KGDH), and pyruvate dehydrogenase (PDH). This route requires elevated cytosolic Ca^2+^ because MCU has a low affinity for Ca^2+^. AcCoA, acetyl coenzyme A; AOA, amino‐acetic acid; Asp, aspartate; Cin, cinnamate; GAPDH, glyceraldehyde‐3‐phosphate dehydrogenase; Glu, glutamate; α‐KG, α‐ketoglutarate; Mal, malate; NAD^+^, oxidized nicotinamide adenine dinucleotide; NADH, reduced nicotinamide adenine dinucleotide; OAA, oxaloacetate; Pyr, pyruvate.

These results led to the conclusion that the long‐known malate–aspartate shuttle may act together with lactate dehydrogenase (LDH) and glycolytic reactions to form a Ca^2+^‐controlled generator of pyruvate (Fig. 1).

Glutamate enters the mitochondria via two different types of transporter families (Fig. 1): the Ca^2+^‐dependent aralar (AGC1, SLC25A12) or its iso‐enzyme citrin (AGC2, SLC25A13) [16, 17, 18], and Ca^2+^‐insensitive glutamate carriers (GC1, SLC25A22, and GC2, SLC25A18) [19, 20]. When mitochondrial respiration is measured with simple glutamate–malate supplementation, both transporter families (AGCs and GCs) are normally active. Unfortunately, there is no specific inhibitor of mitochondrial GCs, which makes it difficult to assess accurately the effect of cytosolic Ca^2+^ on OXPHOS. Consequently, in the present study of the pyruvate generator, we used a fully reconstituted malate–aspartate shuttle (MAS) model [15], which we had previously applied to mitochondria from the mouse heart and whole brain [14].

In preliminary experiments, we found that glutamate‐dependent respiration of isolated rat brainstem mitochondria, unlike that of mitochondria from other brain regions, was surprisingly not activated by the administration of extra‐mitochondrial Ca^2+^. This observation raises the question: Do brainstem mitochondria lack the Ca^2+^‐dependent pyruvate generator, or are other factors masking its presence? To answer this, we examined the Ca^2+^ dependence of maximal mitochondrial respiration using glutamate–malate and pyruvate–malate as substrates in mitochondria isolated from (i) whole rat brain, (ii) seven different rat brain regions, (iii) rat spinal cord, and (iv) two mouse brain areas. This approach allowed us to select two model brain regions with markedly different responses to calcium—the cerebellum (very high Ca^2+^ activation of glutamate‐dependent respiration) and brainstem (very low Ca^2+^ activation of glutamate‐dependent respiration).

Finally, using the fully reconstituted MAS model we demonstrated that the pyruvate generator operates at equally high levels in brainstem mitochondria as in cerebellar mitochondria, even though simple glutamate respiration of rat brainstem mitochondria is not enhanced by Ca^2+^ addition due to the high rate of Ca^2+^‐insensitive glutamate utilization.

## Materials and methods

### Experimental animals

The experiments were performed with 8‐week‐old male Crl:WI(Han) rats from Charles River and with 2‐ to 4‐month‐old male C57BL/6J mice from The Jackson Laboratory. The animals were housed in groups (two rats or four mice per cage) on a 12 h light/12 h dark cycle with *ad libitum* access to water and standard chow.

All experiments were performed in accordance with the ethical guidelines for animals in experiments and were approved by the local animal care committee (Landesverwaltungsamt Sachsen‐Anhalt, Germany, permit ID IPHY/G/01‐1383/16).

### Chemicals and buffers

All chemicals were purchased from Sigma‐Aldrich, except for KCl, HCl, D‐Mannitol, TMPD, diammonium‐dichloride, and Tris‐buffer (Merck) and digitonin (Serva).

The preparation media were designated MES‐A and MES‐B. Both contain 225 mm mannitol, 75 mm sucrose, 20 mm MOPS and 0.5 mm DTT; MES‐A additionally contains 1 mm EGTA, whereas MES‐B contains 0.1 mm EGTA. The pH of both buffers was adjusted to 7.4 with 1 M HCl.

The incubation medium for mitochondria (BIM‐1000) contained 120 mm D‐mannitol, 60 mm KCl, 5 mm KH_2_PO_4_, 5 mm MgCl_2_·6 H_2_O, 40 mm MOPS, 0.1 mm EDTA, and 1000 μm EGTA, pH 7.4. The chelating capacity of EGTA reduces the free Ca^2+^ concentration to ~12 nm, allowing a controlled assessment of Ca^2+^ effects on mitochondrial function.

### Tissue preparation

The animals were euthanized in isoflurane‐anesthesia chamber and then decapitated. The dissected spinal cords and brains were immediately transferred to ice‐cold, EGTA‐rich MSE‐A solution to keep the free Ca^2+^ concentration as low as possible. Individual central nervous system (CNS) regions (cerebellum, brainstem, cortex, corpus callosum, hippocampus, substantia nigra, striatum, and spinal cord) were then isolated and kept on ice‐cold MSE‐A.

### Isolation of mitochondria

Mitochondria were isolated using the method of Kudin et al. [21]. The prepared tissues were first minced with scissors and homogenized in 2 mL of MSE‐A containing 0.05% nagarse (1 mL g^−1^ tissue) using a glass homogenizer. The homogenate was diluted with 5 mL of cold MSE‐A and centrifuged at 2000 ***g*** for 4 min at 4 °C. The supernatant was passed through cheesecloth and centrifuged at 12 000 ***g*** for 10 min at 4 °C. The resulting pellet was re‐suspended in 5 mL MSE‐A containing 0.02% digitonin to permeabilize synaptosomal membranes and then centrifuged again for 10 min at 12 000 ***g***. The final pellet was re‐suspended and gently homogenized in 80 μL MSE‐B. Afterwards, the protein content was determined (see below). The mitochondrial suspensions were stored on ice (4 °C) until respiration measurements were performed.

### Enzymatic measurements

We measured the activity of citrate synthase (CS) [22] and mitochondrial glutamic‐oxaloacetic transaminase (GOT2) [23] in sonicated mitochondrial preparations using standard methods. All measurements were performed at 30 °C with a dual‐wavelength spectrophotometer (Olis‐modernized DW‐2, Spectrophotometer, Bogart, Georgia, USA). The enzymatic assay for GOT2 is based on coupling oxaloacetate (OAA) synthesis by GOT2 to the malate dehydrogenase (MDH) reaction, in which NADH reduces OAA to malate. The rate of NADH consumption (directly proportional to GOT2 activity) was monitored by the decrease in absorbance at 340 nm. The activities of both enzymes are expressed as units per mg of mitochondrial protein.

### Protein determination

Protein concentration was measured with a bicinchoninic acid (BCA) assay kit (Sigma, St. Louis, USA) following the manufacturer's instructions.

### High‐resolution respirometry

Mitochondrial respiration was recorded with an OROBOROS Oxygraph‐2 k (Clark‐type oxygen electrode; Oroboros Instruments, Innsbruck, Austria) at 30 °C. All assays were performed with 0.06 mg mitochondrial protein·mL^−1^ in BIM‐1000 medium, using the multiple‐substrates‐inhibitors protocol described previously [14].

### Statistical analyses

Statistical analyses were carried out with IBM SPSS Statistics (Version 28, New York. USA). Depending on the experimental design, different ANOVA models were applied: one‐way ANOVA with appropriate post hoc test or independent‐samples *t*‐test for pairwise comparisons. A *P* < 0.05 was considered statistically significant. Data are presented as mean ± SEM.

## Results

### Significant differences in Ca^2+^ titration of mitochondrial glutamate respiration in individual regions of the central nervous system

Firstly, we performed a systematic analysis of isolated mitochondria isolated from nine distinct rat CNS regions and two mouse brain regions. Under State 3 conditions (2 mm ADP present in excess), the respiratory rate depends solely on the metabolic oxidation activity of the supplied substrates (glutamate + malate or pyruvate + malate) and on the free Ca^2+^ concentration in the incubation medium. All measurements were made in a medium in which the initial free Ca^2+^ concentration was kept at ~12 nm by the addition of excess EGTA. Free Ca^2+^ levels in the incubation medium were determined fluorometrically in parallel experiments (data not shown).

**Fig. 2:**
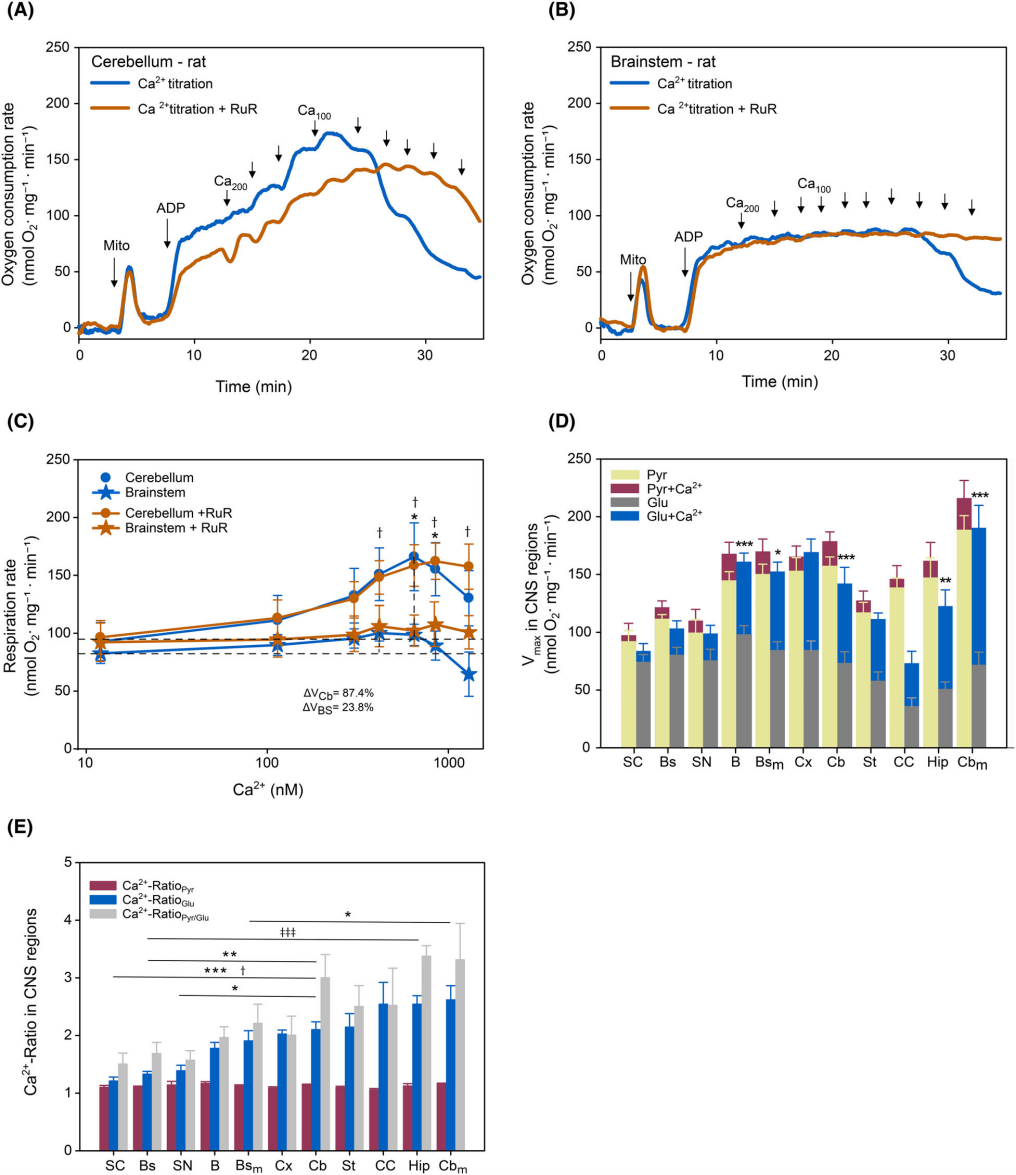
Ca^2+^‐dependent mitochondrial respiration in rat and mouse brain regions. Representative O_2_‐consumption traces (respirograms) obtained during Ca^2+^ titration of isolated rat cerebellar (A) and rat brainstem (B) mitochondria. Mitochondria (0.06 mg protein·mL^−1^) were incubated in BIM‐1000 medium containing 2 mm malate, 10 mm glutamate and 2 mm ADP. Traces were recorded in the absence (blue line) and in the presence of 200 nm ruthenium red (RuR; orang line), an MCU inhibitor. Ca^2+^ was added step‐wise: 200 μm (Ca_200_) followed by 100 μm Ca^2+^ (Ca_100_). (C) Quantification of Ca^2+^‐induced increase in respiration (ΔV) for cerebellum (ΔV_Cb_) and brainstem (ΔV_Bs_). Error bars represent mean ± SEM, *n* = 6 independent experiments per group; blue symbols = without RuR; orange symbols = with 200 nm RuR. Statistical significance was assessed with independent‐samples *t*‐test: versus brainstem in ‘no RuR’ condition, *P < 0.05; versus brainstem in the presence of RuR, ^†^ *P < *0.05. (D) Maximal respiration rates (nmolO_2_·mg^−1^·min^−1^) measured with either 10 mm glutamate +2 mm malate or 10 mm pyruvate +2 mm malate in the absence (gray and yellow bars) or presence (blue and pink bars) of 800 nm Ca^2+^. Data are shown for following CNS regions (rat, *n* = 4–13; mouse, *n* = 3–7). The error bars represents the mean ± SEM. Statistical significance versus corresponding ‘no Ca^2+^’ condition for each substrate was determined by independent‐samples t‐test: *P < 0.05, **P < 0.01, ***P < 0.001. spinal cord (SC), brainstem (Bs), substantia nigra (SN), whole brain (B), cortex (Cx), cerebellum (Cb), striatum (St), corpus callosum (CC), hippocampus (Hip), mouse brainstem (Bs_m_) and mouse cerebellum (Cb_m_). (E) Ca^2+^‐activation ratios. The Ca^2+^‐Ratios (Ca^2+^‐Ratio_Pyr_ and Ca^2+^‐Ratio_Glu_) represent the degree of stimulation produced by extra‐mitochondrial Ca^2+^ when pyruvate or glutamate is used as respiratory substrate. The Ca^2+^‐Ratio_Pyr/Glu_ indicates how much Ca^2+^‐independent glutamate respiration contributes to the maximal possible respiration in the presence of Ca^2+^ with pyruvate as substrate. The ratios were calculated as (V_max +_ Ca^2+^)/V_max_ for each brain region, where V_max_ is the maximal respiration rate without Ca^2+^. Error bars represent mean ± SEM. Statistical significance was assessed with one‐way ANOVA: *P < 0.05, **P < 0.01, ***P < 0.001 for Ca^2+^‐Ratio_Glu_, ^†^ *P < *0.05 for Ca^2+^Rati_Pyr_ and ^‡‡‡^ *P < *0.001 for Ca^2+^‐Ratio_Pyr/Glu_.

To assess the classical (MCU‐dependent) activation of oxidative phosphorylation by Ca^2+^, we performed Ca^2+^ titrations in the presence and in the absence of RuR, an MCU inhibitor. Figure 2A shows a representative Ca^2+^ titration of glutamate‐dependent respiration in rat cerebellar mitochondria. In both the RuR‐free and RuR‐treated preparations, the addition of Ca^2+^ increased the respiration rate, indicating that the response is largely driven by aralar activation through extra‐mitochondrial Ca^2+^. In the absence of RuR, the maximal respiration rate was reached at ~800 nm Ca^2+^ (Fig. 2A,C). At higher Ca^2+^ concentrations above 1 μm (Ca^2+^ overload), conformational changes occur in the MCU, allowing rapid Ca^2+^ influx into matrix and opening of mitochondrial permeability transition pore (mPTP). This leads to collapse of the mitochondrial membrane potential (Δψ), mitochondrial swelling, release of cytochrome c, and finally irreversible loss of mitochondrial function. When RuR was present, slightly higher Ca^2+^ concentrations were required to achieve the maximal rate (Fig. 2A,C), and no overload was observed because the MCU was blocked.

In contrast, Ca^2+^ titration of rat brainstem mitochondria (Fig. 2B,C) produced no significant increase in respiration upon Ca^2+^ addition. As in the cerebellum, RuR prevented Ca^2+^ overload at high Ca^2+^ concentrations, but the overall Ca^2+^‐dependent stimulation remained negligible.

Figure 2D summarizes the maximal respiration rates obtained with pyruvate–malate and glutamate–malate in all CNS regions, both with and without optimal Ca^2+^ levels. In every preparation, pyruvate yielded a higher maximal respiratory rate than glutamate, irrespective of Ca^2+^ supplementation.

The Ca^2+^‐activation ratios (Ca^2+^‐Ratios) are shown in Fig. 2E. Each ratio was calculated as Ca^2+^‐Ratio = Maximum respiration at optimal [Ca^2+^]/Maximum respiration without added Ca^2+^. Ca^2+^‐Ratio_Pyr_ (in the presence of pyruvate) values were nearly identical across all mitochondria (lowest: rat spinal cord = 1.05 ± 0.03; highest: mouse cerebellum = 1.14 ± 0.05).

In contrast, Ca^2+^‐Ratio_Glu_ (in the presence of glutamate) varied markedly (lowest: rat spinal cord = 1.21 ± 0.07; brainstem = 1.33 ± 0.05; substantia nigra = 1.39 ± 0.1; highest: mouse cerebellum = 2.62 ± 0.65; rat hippocampus = 2.54 ± 0.39; corpus callosum = 2.54 ± 1.0). Moderate activations (1.78 ± 0.11–2.14 ± 0.24) were observed in whole brain, mouse brainstem, rat cerebellum, and striatum.

To evaluate the contribution of the Ca^2+^‐insensitive glutamate respiration relative to the maximal possible respiration, we calculated the ratio: pyruvate respiration (with Ca^2+^)/glutamate respiration (without Ca^2+^). This ratio displayed substantial regional variability, ranging from 1.50 ± 0.19 in rat spinal cord to 3.31 ± 0.63 in mouse cerebellum.

### A substrate titration confirms regional differences in Ca^2+^ stimulated, glutamate‐dependent respiration of cerebellar and brainstem mitochondria

The calcium titrations presented above were performed at non‐physiologically low initial Ca^2+^ concentrations and a high, constant concentration of the respiratory substrates. Because substrate concentrations strongly influence enzyme activation, we next performed substrate‐titration experiments at fixed low Ca^2+^ level (12 nm) and fixed high Ca^2+^ (800 nm), both in the presence and absence of RuR.

**Fig. 3:**
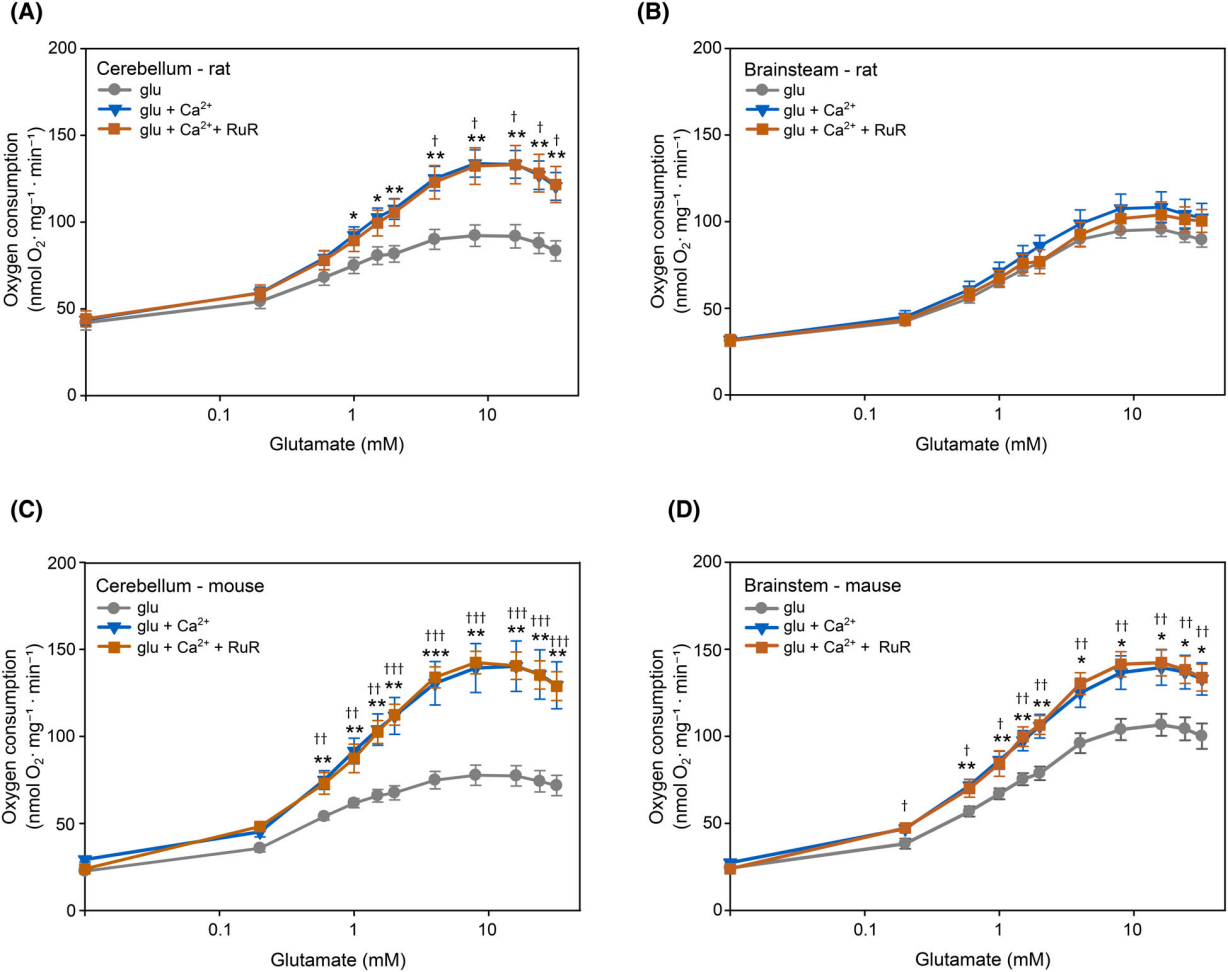
Glutamate‐titration curves for cerebellar and brainstem mitochondria. Mitochondria (0.06 mg protein·mL^−1^) isolated from rat cerebellum (A), rat brainstem (B), mouse cerebellum (C), and mouse brainstem (D) were incubated in BIM‐1000 medium containing 2 mm ADP, 2 mm malate. Experiments were performed without Ca^2+^ and without RuR (gray line), in the presence of 800 nm Ca^2+^ with (orange line) and without (blue line) 200 nm RuR. Glutamate was added step‐wise from 0 to 32 mm, and oxygen consumption was recorded after each addition. The error bars represent the mean ± SEM. Statistical significance was determined using independent‐samples *t*‐test versus the control condition (‘no Ca^2+^, no RuR’); for the Ca^2+^ condition: *P < 0.05, **P < 0.01, ***P < 0.001; and for the Ca^2+^ + RuR condition: ^†^ *P < *0.05, ^††^ *P < *0.01,^†††^ *P < *0.001, *n* = 7 independent experiments per each condition.

Figure 3A,B shows the glutamate‐titration curves of rat cerebellar and brainstem mitochondria, respectively. Under these conditions, brainstem mitochondria displayed a very modest activation of respiration by extra‐mitochondrial Ca^2+^. The maximal increase in respiration rate at an optimal Ca^2+^ concentration (800 nm) relative to the control (12 nm) was 12.71 ± 7.62 nmolO_2_·mg^−1^·min^−1^ in brainstem mitochondria and 41.58 ± 9.86 nmolO_2_·mg^−1^·min^−1^ in cerebellar mitochondria. A similar pattern was observed in mouse brain tissue. In the brainstem, the high‐Ca^2+^ medium raised the respiration rate by 32.95 ± 9.61 nmolO_2_·mg^−1^·min^−1^, whereas in the cerebellum, the increase was 62.98 ± 11.09 nmolO_2_·mg^−1^·min^−1^ (Fig. 3C,D).

In all cases, treatment with RuR had no effect on Ca^2+^‐stimulated respiration. The Michaels–Menten constant (*K*_m_) for glutamate in both brain regions of rat and mouse mitochondria ranged from 0.3 mm to 0.9 mm and did not depend on Ca^2+^.

### Complete reconstitution of the MAS confirms strong Ca^2+^‐stimulated, MAS‐dependent respiration in mitochondria from both the cerebellum and the brainstem

**Fig. 4:**
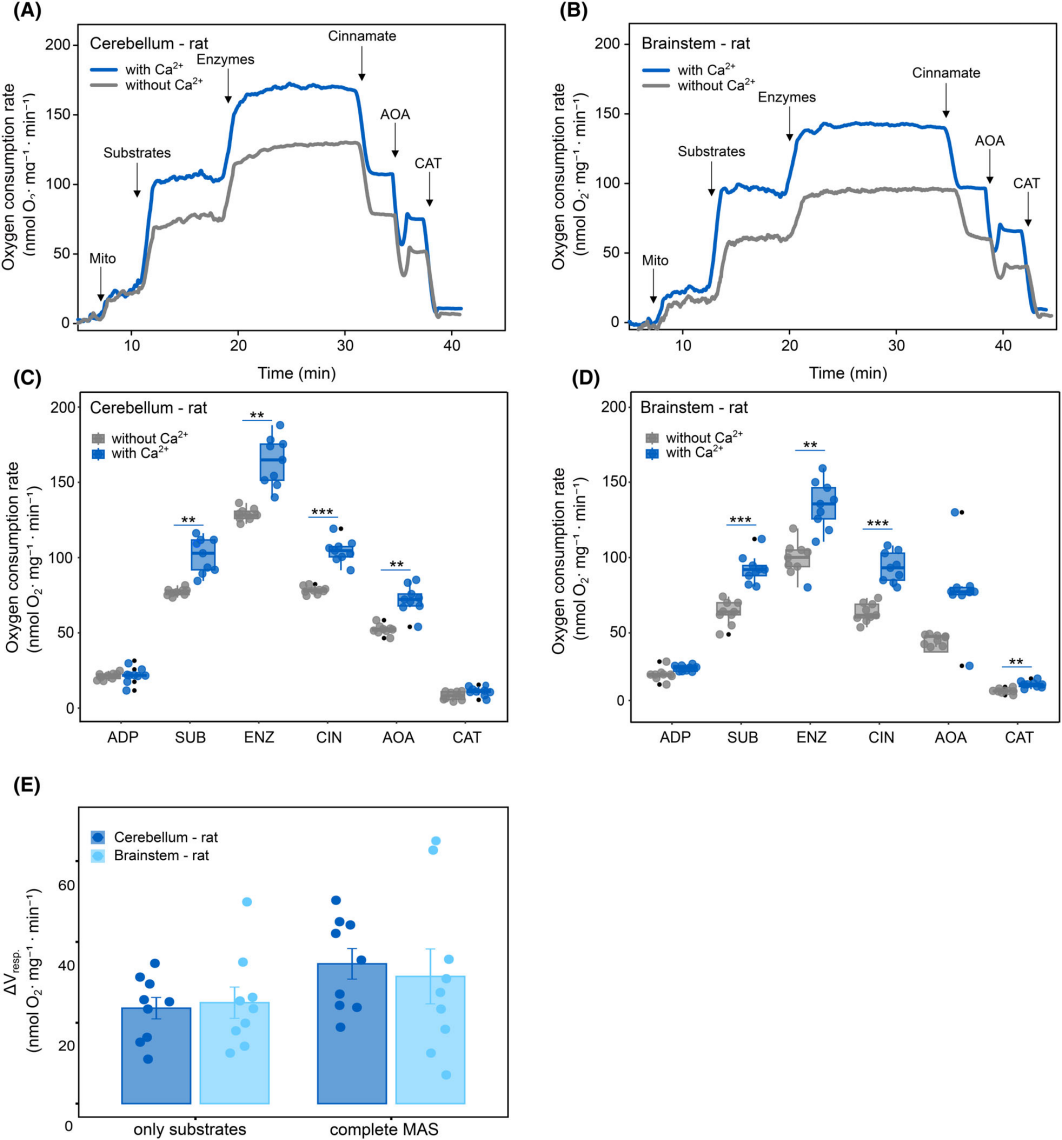
Extra‐mitochondrial Ca^2+^ amplifies respiration after complete malate–aspartate shuttle (MAS) reconstitution. Representative respiration traces from rat cerebellar (A) and brainstem (B) mitochondria (0,06 mg protein·mL^−1^) incubated in BIM‐1000 medium with 2 mm ADP, 2 mm malate, 5 mm lactate and 250 μm NADH. Recordings were made without (gray line) and with 800 Ca^2+^ (blue line). After establishing equilibrium, the MAS substrates were added: 2 mm glutamate, 2 mm aspartate and 2 mm α‐oxoglutarate following by cytosolic MAS enzymes—5 U·mL^−1^ LDH, MDH, and GOT. Finally, inhibitors 1 μm cinnamate (CIN), 2 mm amino‐oxiactetate (AOA) and 2.5 μm carboxyatractyloside (CAT) were added. Quantitative analysis of respiration rates after full MAS reconstitution in cerebellar (C) und brainstem (D) mitochondria. Box plots show median (center line), inter‐quartile range (box), whiskers (minimum–maximum), individual data points (colored dots, *n* = 9) and outliers (black dots). Statistical significance was determined by independent‐samples t‐test for the comparison between Ca^2+^ free and Ca^2+^‐present conditions; **P < 0.01,***P < 0.001. (E) Absolut Ca^2+^‐induced increase in respiration (ΔV_resp_.) calculated as V (800 nm Ca^2+^) – V (no Ca^2+^), where V is maximal respiration. Error bars represent mean ± SEM, colored dots represent individual data points (*n* = 9 per group).

Because the experiments described above do not reproduce physiological conditions (the extra‐mitochondrial arm of the MAS is absent and pyruvate cannot be generated from lactate; see Fig. 1), we employed a fully reconstituted MAS model, which is described in detail in our previous publications [14, 15]. This approach allows a direct assessment of the MAS contribution to substrate delivery for OXPHOS (Fig. 4A,B).

When cerebellar and brainstem mitochondria were incubated with lactate, malate, NADH, and ADP in a medium containing a very low or a high Ca^2+^ concentration, respiratory activity was minimal because essential substrates were missing. Adding the full set of MAS substrates (glutamate, aspartate, and α‐ketoglutarate; NAD^+^, oxaloacetate, and pyruvate were not added) produced a pronounced increase in respiration in cerebellar 101.21 ± 3.88 nmolO_2_·mg^−1^·min^−1^ and brainstem mitochondria 92.46 ± 3.15 nmolO_2_·mg^−1^·min^−^1, both in the presence of Ca^2+^. Subsequent addition of the cytosolic MAS enzymes (LDH, MDH, and GOT) caused a further strong rise in the respiratory rate in both mitochondrial preparations (163.38 ± 5.38 nmolO_2_·mg^−1^·min^−1^ and 134.90 ± 5.19 nmolO_2_·mg^−1^·min^−1^, respectively). The increase reflects the enzymatic conversion of lactate to pyruvate and subsequent import of pyruvate into the matrix for oxidation. The addition of cinnamate (CIN), a specific inhibitor of the mitochondrial pyruvate carrier, reduced respiration back to the level observed before the MAS enzymes were added (104.50 ± 2.55 nmolO_2_·mg^−1^·min^−1^ and 93.47 ± 3.36 nmolO_2_·mg^−1^·min^−1^, respectively). Inhibition of GOT with amino‐acetic acid (AOA) caused an additional decline in respiration because the production of aspartate and α‐ketoglutarate (both inside and outside the mitochondria) was blocked, thereby halting the MAS cycle. Finally, addition of carboxyatractyloside (CAT), an inhibitor of the adenine‐nucleotide translocase, completely abolished oxidative phosphorylation.

Quantitative analysis of the MAS reconstitution experiments showed that the mitochondrial respiration from both rat brain regions was activated to a similar extent by the combined addition of substrates, enzymes, and Ca^2+^ (Fig. 4C,D). This contrasts with the simple glutamate‐dependent respiration assay, in which rat brainstem mitochondria displayed no Ca^2+^ dependency of respiration (see Fig. 2C).

The absolute increase in respiration rate (ΔV_resp_.), expressed as the difference between the rates measured in the absence and presence of Ca^2+^, is shown in Fig. 4E. The values are almost identical: substrate‐only condition—23.61 ± 2.65 nmolO_2_·mg^−1^·min^−1^ (cerebellum) vs. 24.47 ± 3.86 nmolO_2_·mg^−1^·min^−1^ (brainstem), complete MAS condition—34.58 ± 3.78 nmolO_2_·mg^−1^·min^−1^ (cerebellum) vs. 31.47 ± 6.79 nmolO_2_·mg^−1^·min^−1^ (brainstem mitochondria). This is possible only when MAS activity is comparable in both mitochondrial preparations, as has been shown for GOT2 (Table S1).

## Discussion

The observed ability of extra‐mitochondrial Ca^2+^ to stimulate glutamate‐dependent respiration in isolated brain mitochondria, together with the lack of significant activation of pyruvate respiration by calcium, led to the discovery of the Ca^2+^‐stimulated pyruvate generator (‘mitochondrial gas pedal’) [14, 15]. In the present study, we have shown that mitochondria from different regions of the CNS exhibit varying sensitivity of glutamate‐supported respiration to extra‐mitochondrial Ca^2+^. These findings raise the question of whether the pyruvate generator is absent or less active in some brain structures, or whether the measurement of its activity is affected by experimental conditions. How is the supply of substrates for OXPHOS regulated in these brain areas?

Using a simple measurement of glutamate‐dependent respiration, with and without Ca^2+^ addition and in the presence or absence of RuR, we have shown that calcium significantly increases respiration rate of rat cerebellar, but not rat brainstem mitochondria. These experiments showed that, in cerebellar mitochondria, glutamate‐dependent respiration (without Ca^2+^) accounts for only ~40% of maximal pyruvate‐driven respiration (with Ca^2+^), and the respiration rate can be significantly increased by the addition of Ca^2+^. This increase is mainly due to the rapid transport of glutamate into the matrix by the Ca^2+^‐sensitive aralar. Inside the mitochondrial matrix, glutamate is converted to α‐ketoglutarate by the mitochondrial glutamate‐oxaloacetate transaminase, and together with malate that is also imported, provides reducing equivalents for the electron‐transport chain, or it can be directly fed into the TCA cycle. In fact, extra‐mitochondrial Ca^2+^ strongly stimulates glutamate respiration in RuR‐treated cerebellar mitochondria, where the classical OXPHOS‐activating mechanism must be inactive. These results support the hypothesis that OXPHOS can be regulated by extra‐mitochondrial Ca^2+^ as well as by the traditional matrix‐Ca^2+^ pathway.

Previously, the aspartate–glutamate exchanger was shown to increase metabolite transport and ATP production in response to cytosolic Ca^2+^ elevations [24, 25]. It should be noted, however, that MAS also operates in the absence of Ca^2+^‐mediated aralar activation, albeit at a low basal level [26]. In contrast, in brainstem mitochondria where glutamate respiration (without Ca^2+^) represents more than 65% of the maximal pyruvate respiration (with Ca^2+^), the addition of calcium cannot significantly raise the respiration rate because OXPHOS is already operating near its maximal capacity, even if the aralar is stimulated by extra‐mitochondrial Ca^2+^. The differing sensitivity of glutamate‐supported respiration to Ca^2+^ in the brainstem versus cerebellar mitochondria was also confirmed in glutamate‐titration experiments performed at a constant Ca^2+^ concentration. At the optimal glutamate concentration, extra‐mitochondrial Ca^2+^ increased respiration in cerebellar mitochondria by ~45% and in the brainstem mitochondria by only ~13%, even when the mitochondria were incubated with RuR. Therefore, is the pyruvate generator less active in brainstem mitochondria? The question was addressed with experiments using a fully reconstituted MAS model. The experimental system demonstrated that extra‐mitochondrial Ca^2+^ can stimulate glutamate‐supported respiration in the brainstem to a level comparable with that in the cerebellum. Immediately after the addition of cytosolic MAS enzymes, a pronounced increase in respiration rate was observed, reflecting the rapid onset of pyruvate formation in both cerebellar and brainstem mitochondria. This finding confirms the presence and activity of the Ca^2+^‐dependent pyruvate‐generating pathway in both brain regions.

It should be noted that, in these experiments, the Ca^2+^‐independent glutamate transporter is also active, although transport via the Ca^2+^‐stimulated aralar is energetically more favorable. Glutamate import through aralar is driven by four gradients: glutamate, H^+^, aspartate, and the membrane potential (Δψ) [15, 16], whereas the Ca^2+^‐independent carrier relies on only two gradients (H^+^ and glutamate) [19].

Using this method, we have previously shown that the pyruvate generator also operates in heart, whole‐brain mitochondria from MCU‐KO mice, intact synaptosomes and working rat heart, indicating that this mechanism does not require Ca^2+^ entry into the mitochondrial matrix [14].

Why, during a simple measurement of glutamate‐dependent respiration, was no stimulating effect of Ca^2+^ observed in brainstem mitochondria despite the presence of functional aralar? The most plausible explanation is a lower capacity of the MAS in brainstem mitochondria, which reflects the cellular composition of this brain region. Aralar is highly expressed in neurons but is much less abundant in oligodendrocytes and astrocytes [27, 28, 29] (Table S2). In studies on unstimulated, intact neurons, mitochondrial respiration at physiological glucose concentrations was shown to depend on the aralar‐MAS, indicating that its main function is to shuttle NADH and deliver pyruvate to the matrix. Consistently, the maximal respiration rate of aralar‐KO neurons is reduced by ~46% [26]. Del Arco and colleagues demonstrated that, in neurons, the MAS plays a major role, especially under small‐to‐submaximal workloads [30].

A second important factor might be the differential expression of GCs in the plasma and mitochondrial membranes of brain cells [31, 32]. Berkich and colleagues reported that GC1 is expressed at much higher levels in mitochondria derived from astrocytes and other glial cells than in neurons [31]. Data from the Human Protein Atlas clearly show that GC2 dominates in the glial‐cell‐rich brainstem (Table S2). This particular feature of glial‐cell mitochondria might be important for efficient glutamate sequestration in the brain, avoiding spillover of this neurotransmitter. Consequently, in the brainstem where white matter and glial cells predominate, mitochondrial GC activity is expected to exceed that of aralar, so that glutamate is taken up mainly via the Ca^2+^‐independent pathway. In this scenario, further oxidation of glutamate in the matrix is carried out by GDH and α‐KGDH, providing maximal glutamate‐supported OXPHOS efficiency. Calcium supplementation does activate aralar, but the conversion of glutamate to α‐ketoglutarate by mitochondrial GOT2 cannot further increase the respiration rate, even though GOT2 activity was comparable in cerebellar and brainstem mitochondria (Table S1). The respective contributions of aralar and the glutamate carriers to glutamate transport and its oxidation in mitochondria have been discussed in detail by McKenna and colleagues [9]. Nevertheless, further research is needed to precisely determine which cells in specific brain structures express mitochondrial GCs and what impact this may have on glutamate utilization in those brain regions.

In summary, we can therefore conclude that the pyruvate generator stimulated by extra‐mitochondrial Ca^2+^ is universally present throughout all regions of the rat brain. A simple measurement of glutamate‐dependent respiration is not sufficient to confirm the presence or its activity because of inherent limitations of *in‐vitro* assays. The Ca^2+^‐dependent supply of substrates via the MAS to OXPHOS is modulated not only by the specific cellular composition and the expression levels of mitochondrial transporters in each brain structure, but also by numerous subtle factors that are difficult to reproduce in experiments with isolated mitochondria.

## Conflicts of interest

The authors declare no conflicts of interest.

## Author contributions

GD‐V and FNG were involved in writing the manuscript, data validation, conceptualization, and editing the manuscript. NK, MK, and TG performed experiments and analyzed the data. ZG and FNG designed experiments. SV and WSK made manuscript revisions.

## Supporting information


**Table S1.** Oxaloacetic transaminase and citrate synthase activities in rat cerebellar and brainstem mitochondria.
**Table S2.** Expression of glutamate transporters in human brain obtained by RNA sequencing.

## Data Availability

The data that support the findings of this study are available from the corresponding author upon reasonable request. Data on the expression of glutamate transporters in the brain are publicly available on the Human Protein Atlas website (https://www.proteinatlas.org/). Additional supporting information may be found online in the supporting information section at the end of the article.
