# Supplementary material for: The pyruvate generator is a common phenomenon in mitochondria from different rat and mouse brain regions
Source: FEBS Lett. 2026 May 23;600(12):1770–81. doi: 10.1002/1873-3468.70370 (PMC13284805; doi:10.1002/1873-3468.70370)
Supplement: Supplementary file 1 — Table S1. Oxaloacetic transaminase and citrate synthase activities in rat cerebellar and brainstem mitochondria. Table S2. Expression of glutamate transporters in human brain obtained by RNA sequencing. [file FEB2-600-1770-s001.docx]

**Supplementary Table S1**. **Oxaloacetic transaminase** and citrate synthase activities in rat cerebellar and brainstem mitochondria.

| Brain region | Citrat synthase (CS)  (n=3) | **Mitochondrial Glutamic-Oxaloacetic Transaminase (GOT2)**  (n=3) | GOT2/CS |
| --- | --- | --- | --- |
| Cerebellum | 0.78 ± 0.03 | 1.22 ± 0.09 | 1.56 ± 0.12 |
| Brainstem | 0.77 ± 0.03 | 1.19 ± 0.06 | 1.54 ± 0.14 |

Enzymatic measurements of CS and GOT2 were performed as described in methods. Activity of CS and GOT2 are express as U·mg^-1^ protein. Data are presented as mean ± SD (n = 3 independent experiments).

**Supplementary Table S2**. Expression of glutamate transporters in human brain obtained by RNA sequencing.

| Transporter | Gene name | Human brain* |  |  |
| --- | --- | --- | --- | --- |
|  |  | Midbrain | Pons | Cerebellar cortex |
| AGC1 (aralar) | SLC25A12 | 9.2 | 12.9 | 17.7 |
| AGC2 (citrin) | SLC25A13 | 11.2 | 17.5 | 9.5 |
| GC1 | SLC25A22 | 75.6 | 101.0 | 78.6 |
| GC2 | SLC25A18 | 90.3 | 53.6 | 32.0 |

* data from human protein atlas (<https://www.proteinatlas.org/>) in nTPM
